# Identification of a Selective G1-Phase Benzimidazolone Inhibitor by a Senescence-Targeted Virtual Screen Using Artificial Neural Networks^1^^2^

**DOI:** 10.1016/j.neo.2015.08.009

**Published:** 2015-10-19

**Authors:** Alan E. Bilsland, Angelo Pugliese, Yu Liu, John Revie, Sharon Burns, Carol McCormick, Claire J. Cairney, Justin Bower, Martin Drysdale, Masashi Narita, Mahito Sadaie, W. Nicol Keith

**Affiliations:** ⁎ Institute of Cancer Sciences, University of Glasgow, Wolfson Wohl Cancer Research Centre, Garscube Estate, Switchback Road, Bearsden, Glasgow G61 1QH, UK; † Cancer Research UK Beatson Institute, Garscube Estate, Switchback Road, Bearsden, Glasgow G61 1BD, UK; ‡ University of Cambridge, Cancer Research UK Cambridge Institute, Li Ka Shing Centre Robinson Way, Cambridge, CB2 0RE, UK; § Kyoto University, Graduate School of Biostudies, Yoshidakonoe-cho, Sakyo-ku Kyoto 606-8501 Japan

## Abstract

Cellular senescence is a barrier to tumorigenesis in normal cells, and tumor cells undergo senescence responses to genotoxic stimuli, which is a potential target phenotype for cancer therapy. However, in this setting, mixed-mode responses are common with apoptosis the dominant effect. Hence, more selective senescence inducers are required. Here we report a machine learning–based *in silico* screen to identify potential senescence agonists. We built profiles of differentially affected biological process networks from expression data obtained under induced telomere dysfunction conditions in colorectal cancer cells and matched these to a panel of 17 protein targets with confirmatory screening data in PubChem. We trained a neural network using 3517 compounds identified as active or inactive against these targets. The resulting classification model was used to screen a virtual library of ~ 2M lead-like compounds. One hundred and forty-seven virtual hits were acquired for validation in growth inhibition and senescence-associated β-galactosidase assays. Among the found hits, a benzimidazolone compound, CB-20903630, had low micromolar IC50 for growth inhibition of HCT116 cells and selectively induced senescence-associated β-galactosidase activity in the entire treated cell population without cytotoxicity or apoptosis induction. Growth suppression was mediated by G1 blockade involving increased p21 expression and suppressed cyclin B1, CDK1, and CDC25C. In addition, the compound inhibited growth of multicellular spheroids and caused severe retardation of population kinetics in long-term treatments. Preliminary structure-activity and structure clustering analyses are reported, and expression analysis of CB-20903630 against other cell cycle suppressor compounds suggested a PI3K/AKT-inhibitor–like profile in normal cells, with different pathways affected in cancer cells.

## Introduction

Cellular senescence in normal cells is an irreversible cell cycle arrest which is involved in cellular aging and tissue maintenance, and which is induced by critically shortened telomeres at the end of replicative lifespan. Oxidative damage and oncogene activation accelerate both telomere shortening and senescence induction [1]. Therefore, senescence is considered to be a barrier to tumorigenesis which cancer cells must bypass to acquire a transformed phenotype [2,3].

Many cancer cells retain the capacity to undergo senescence-like growth arrest in response to agents including chemotherapeutics and ionizing radiation in addition to many targeted agents [4]. Hence, despite inactivation of some key pathways, many tumor cells retain the ability to exit the cell cycle under appropriate treatments. Thus, latent senescence signaling may persist in tumors [5].

There is substantial interest in senescence induction as a therapeutic outcome in cancer. However, senescence involves multiple processes including telomere homeostasis, DNA damage and inflammatory signaling, chromatin regulation, and metabolism [6,7]. Interaction of these with the diverse mutational backgrounds of cancer cells adds further complexity in attempting to define the best targets for therapeutic intervention. It seems likely that a spectrum of senescence-like responses is possible in cancer cells depending on induction agent and signaling environment [8,9].

Given limitations in current knowledge, phenotypic screening is attractive both for compound and pathway discovery focused on senescence [10–12]. Suitable phenotypic markers for assay development include p21 and p16 levels, the senescence-associated secretory phenotype, senescence-associated β-galactosidase (SA-β-gal) staining, senescence-associated heterochromatin foci, and altered morphology [1]. However, although many agents elicit senescence, responses obtained are often restricted to subsets of cells, with apoptotic cell death dominant [13].

To evaluate senescence induction as an anticancer modality will require identification of senescence agonists which are substantially more selective than currently available tools [14]. Without detailed knowledge of targets, the screening challenge is not simply identification of compounds which can cause senescence; rather, stratification of the most selective compounds among many expected partial actives is critical. Identification of enriched libraries would be beneficial before initiating a screening campaign. We reasoned that virtual screening might identify such an enriched set.

Ligand-based virtual screening is of increasing interest in the construction of activity models, ranging from well-defined target binding studies [15] to more complex scenarios such as modeling of experimental microsomal stability results [16], and a wide variety of platforms and datasets are now available [17]. Another major goal is to identify new compounds with activity against a given target based on feature recognition [18].

In either case, abstraction of chemical structure information into a set of numerical descriptors is critical. These must provide detailed representation of the chemical and property space for a given compound set [19]. An assumption is that a relation can be made between these “fingerprints” and a classifier (active/inactive) or known quantity such as IC50. Machine learning methods such as neural networks [18,20] or support vector machines [21,22] provide a powerful approach. Feature recognition rules are learned from a training set with known activity; trained models are then simulated against a new compound set of unknown activity.

Here we report a virtual screen using an artificial neural network ensemble trained by the scaled conjugate gradient descent method [23] using compounds identified from pooled PubChem screens [24,25] against a panel of senescence-related targets. Targets were selected by matching available screens to cellular “process networks profiles” obtained by functional enrichment analysis of expression data in colorectal cancer cells with induced telomere dysfunction. The trained ensemble was used to classify a library of around 2M lead-like compounds, leading to identification of a benzimidazolone compound with low micromolar IC50 which selectively induces G1 blockade and SA-β-gal without causing apoptosis. Preliminary structure/activity relationships (SARs) and clustering studies are reported.

## Results

### Identification of a Senescence-Associated Protein Target Panel

Ad-hTR-mut is an adenoviral vector harboring mutant telomere template sequence [2,26]. Telomerase-dependent reverse transcription in cancer cells incorporates mutant sequence in the telomeres of infected cells, causing rapid telomere damage signaling. This provides a highly selective way to induce telomere dysfunction and cellular senescence.

**Figure 1:**
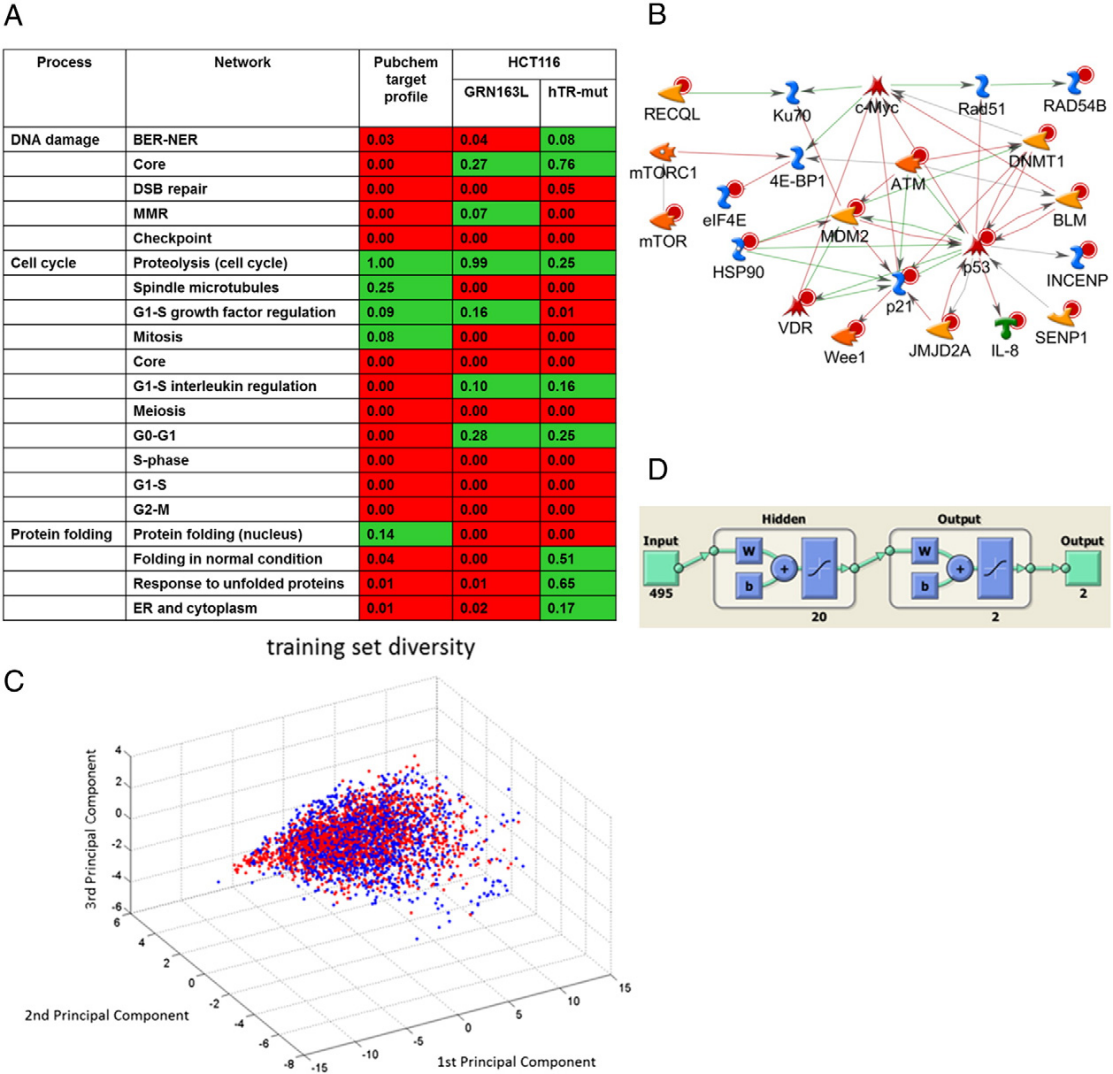
Development of a senescence-targeted virtual screen. (A) Expression microarray data from colorectal cancer cells were used to generate process network profiles of induced telomere dysfunction. Top scoring processes are shown. The complete profile is given in Supplementary Figure S1. Processes in red scored as significant. Numbers in each cell are the hypergeometric *P* value for each gene list against each process network. The main processes affected were matched to targets involved in those processes for which confirmatory screens were available in PubChem. (B) Interactions in the senescence-associated target panel network identified by direct-interactions network building in GeneGo. (C) Diversity of the 3517-compound training set. Principal component analysis was performed on the 495 selected chemical descriptors, and projections on the first three principal components were visualized in Matlab. Actives are shown in blue; inactives are shown in red. (D) Structure of the trained networks. A 10-network ensemble was used for the virtual screen.

To identify pathways associated with telomere dysfunction and senescence, we performed expression profiling and pathway analysis [27–29] on HCT116 colorectal cancer cells infected with Ad-hTR-mut or treated in long-term culture with telomerase inhibitor GRN163L [30,31]. “Process network profiles” were generated by pathway enrichment analysis against 169 curated networks from the MetaCore database [27] covering 23 top-level processes (Supplementary Table S1). Heat maps were generated based on significance of each network to visualize overall significance of each process (Figure 1*A* and Supplementary Figure S1). The most significant enrichments of differentially expressed genes in response to telomere targeting agents were on networks involved in DNA damage, cell cycle, and protein folding.

We hypothesized that targets involved in these telomere dysfunction processes would be good candidate targets for senescence induction. We therefore sought to identify a target group with known involvement in these processes and for which confirmatory (dose-response) screening results were available within the PubChem bioassay database [24,25]. In searching available screens, we identified 17 candidate protein targets with relevance to these processes and with associated confirmatory screens (Table 1).

Enrichment analysis on this target list confirmed close involvement of the panel in the same process networks identified as significantly affected by telomere targeting (Figure 1*A* and Supplementary Figure S1). We also performed shortest-paths analysis of the target panel in MetaCore to determine functional relations between these targets (Figure 1*B*). These targets participate in a closely connected direct interactions network, indicating the close interplay between diverse processes in senescence regulation.

### Development of a Senescence-Targeted Virtual Screen

The overall neural network optimization workflow is shown in Supplementary Figure S2. To develop the classifier, compound sets associated with each identified PubChem bioassay were merged into active and inactive pools (Table 1). As classification models can be affected by an initial unbalance in the data, we aimed at retaining similar compound numbers in each list. Similarity filters were applied to reduce the size of very large inactive lists using ChemOffice. The pooled lists were cleaned, duplicates were removed, and a molecular weight filter was applied (150 < MW < 700). The actives and inactives were then merged to generate the training set, and duplicates arising after the merge were excluded. The more balanced training set contained 3924 compounds of which 1859 were active against the targets panel and 2065 were inactive.

For each compound, 2780 descriptors were generated. These included 729 1D/2D descriptors, 880 PubChem fingerprints and 1024 CDK extended fingerprints obtained using PaDel, and 147 pharmacophore fingerprints obtained using PowerMV [32,33]. An initial parameter scan was performed on this training set to identify the classifier performance when learning rule and neuron number were varied (Supplementary Figure S3). The best initial parameter set (20 neurons, 1 hidden layer, with learning by scaled conjugate descent) gave 73.8% classification accuracy with Cohen’s *κ* = 0.51 [34] in 10-fold cross-validation. To improve the performance, we excluded the compounds that were most consistently misclassified under these parameters (Mean Square Error > 0.4 excluded). A total of 3536 compounds were retained, and a features selection protocol (see methods) was performed on the descriptor set for these. Nineteen compounds were not correctly recognized by the feature selection software and were excluded.

**Figure 2:**
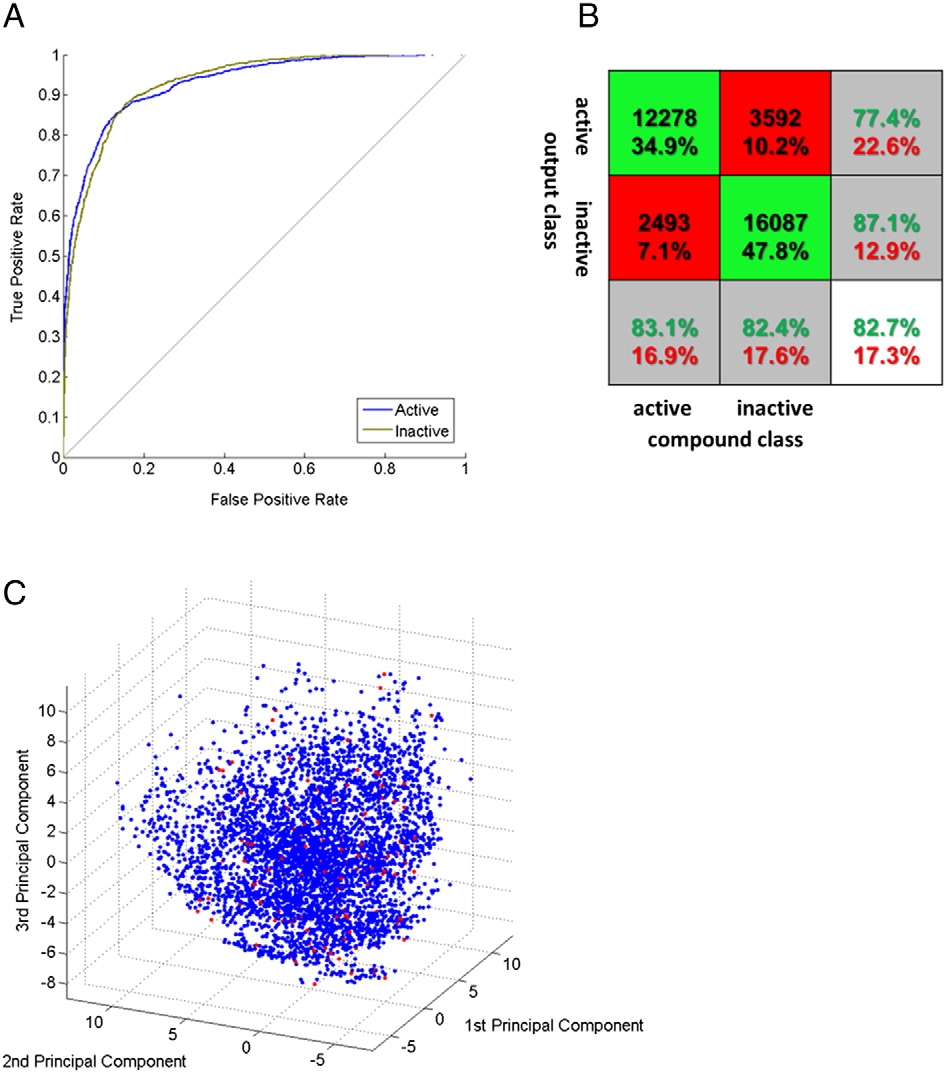
Performance of the network ensemble and virtual screening results. (A) Receiver operating characteristic plot of the performance of 1 of the 10 networks in the trained ensemble showing results for each output neuron. One neuron each classified active or inactive compounds. (B) Summed confusion matrix for the 10-network classifier. Numbers represent total compound number and percentage of the training set falling in each quadrant as classified across all networks. Cohen’s *κ* = 0.65 for the ensemble. (C) Principal component analysis of filtered virtual screening hits (total set in blue) and compounds selected after clustering on 3D pharmacophores (red). Principal component analysis was performed in Matlab on 3D pharmacophores extracted using Canvas.

We retained 495 descriptors for the final 3517 compound set. Principal component analysis on these descriptors is shown in Figure 1*C*, illustrating good overlap between the active and inactive chemical spaces. Parameter scanning was again performed on this data with 10-fold cross-validation. Best accuracy (Supplementary Figure S4) was obtained with 2-layer networks having 20 neurons in the hidden layer with 2 output neurons (Figure 1*D*) trained by scaled conjugate gradient descent [23]. We finally selected an ensemble of 10 networks trained using the optimal parameter, compound, and descriptor sets. A representative receiver operating characteristic plot for one of these is shown in Figure 2*A*. The high area under the curve of each output neuron indicates excellent classification of both active and inactive compounds. The overall sensitivity and specificity of the entire panel were 83.1% and 82.4%, respectively, for the “hit” output neuron (Figure 2*B*). Overall accuracy was 82.7%, and Cohen’s *κ* = 0.65 indicated very good classification performance [35].

We used the trained ensemble to screen 2,086,587 structures with weighting for specificity by application of a cutoff of 0.95 on the active output neuron and 0.05 on the inactive output neuron, resulting in 17,278 virtual hits. To prioritize these, *in silico* physicochemical and ADMET filters were applied (Supplementary Table S2). A total of 4929 compounds remained after filtering, and these were clustered on 3D pharmacophore fingerprints. A final set of 147 for cell-based screening was obtained by sampling from these clusters (Figure 2*C*; SMILES structures are given in Supplementary File 1).

### Identification of a Highly Selective Benzimidazolone SA-β-gal Inducer

**Figure 3:**
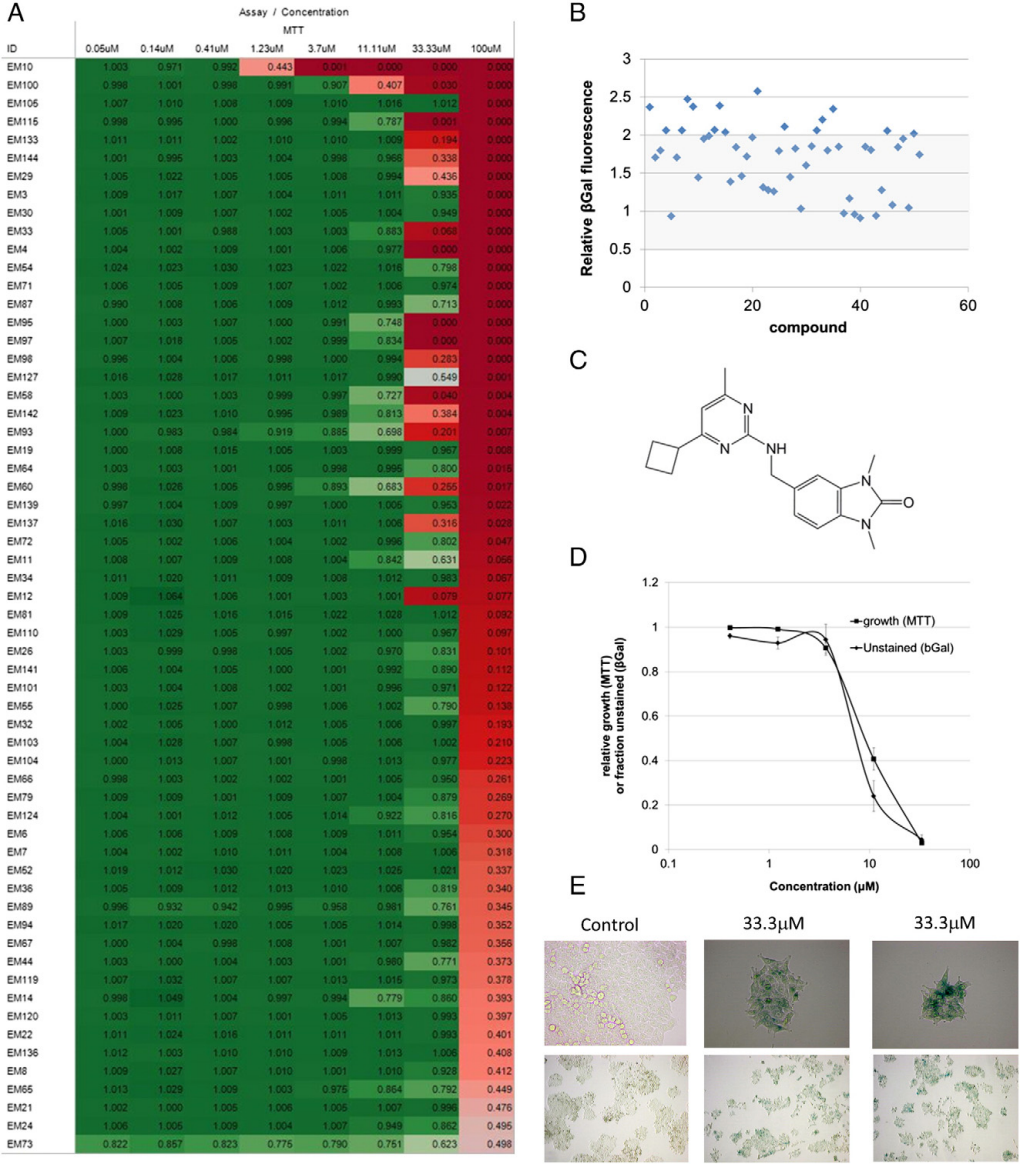
Cell-based screening results and identification of CB-20903630. (A) MTT cell growth inhibition results for compounds showing confirmed dose-dependent inhibition with IC50 < 100 μM. Heat maps were visualized in Tableau desktop. Numbers in each cell represent mean fold of control for each concentration of compound. Mean ± SEM of three experiments. (B) Fluorometric SA-β-gal on the 50 most potent MTT hits. Results are fold of vehicle-treated control. Two-fold activation of fluorescent signal was chosen as cutoff. Mean ± SEM of two experiments. (C) Structure of CB-20903630. The structure was confirmed by nuclear magnetic resonance, and purity was > 95% by liquid chromatography. (D) Growth inhibition and SA-β-gal population-staining dose responses for CB-20903630. To clarify the shared dose response, data shown for SA-β-gal are unstained cells at each dose (1 minus SA-β-gal positive). Mean ± SEM of three experiments (MTT) or two experiments (SA-β-gal). (E) Representative micrographs showing SA-β-gal staining in untreated HCT116 cells or cells treated at 33.3 μM.

The 147 compounds were first tested in MTT (3-(4,5-Dimethylthiazol-2-yl)-2,5-Diphenyltetrazolium Bromide) cell viability assays in HCT116 cells at 100 μM. Ninety-two compounds showed at least 1.5-fold growth inhibition and were taken into dose-response treatments (Supplementary Figure S4). Sixty of the 92 compounds showed confirmed growth inhibition with IC50 < 100 μM (Figure 3*A*), and the top 50 of these with > 60% growth suppression at 100 μM were tested in a fluorometric SA-β-gal assay. Each compound was tested initially at a single dose determined from the MTT results to be the minimum concentration which achieved maximum inhibition within the range tested. For example, this was 3.7 μM for compound EM10 and 33.3 μM for EM100 (Figure 3*A*, *top two rows*).

The fluorometric SA-β-gal results are given in Figure 3*B*. In our hands, the fluorometric assay has log-linear relation with the proportion of HCT116 cells staining positive for SA-β-gal in the standard staining assay in the range up to ~ 25% positive cells at ~ 1.5-fold fluorescence induction (Supplementary Figure S6). We therefore imposed a cutoff of two-fold induction of signal relative to untreated cells. Fifteen compounds achieved greater than two-fold induction and were tested in colorimetric SA-β-gal staining dose responses.

The most potent effects were observed in both assays with EM100. SA-β-gal induction closely mirrored the inhibition of growth by MTT for this compound (MTT IC50, 9.6 μM; SA-β-gal EC50, 8.3 μM) (Figure 3*D*). EM100 is the ChemBridge compound 20903630 (Figure 3*C*, hereafter referred to as CB-20903630). Interestingly, at high concentration (33.3 μM), the compound was able to elicit detectable SA-β-gal staining in almost all cells against a background of extremely low (< 1%) staining in untreated control cells (Figure 3*E*). Furthermore, staining in the entire population was achieved without observable loss of attachment, suggesting little or no cell death. Therefore, CB-20903630 appeared highly selective in inducing a key senescence marker.

### Structure-Activity and Cell Cycle Inhibition Effects of Compound CB-20903630

**Figure 4:**
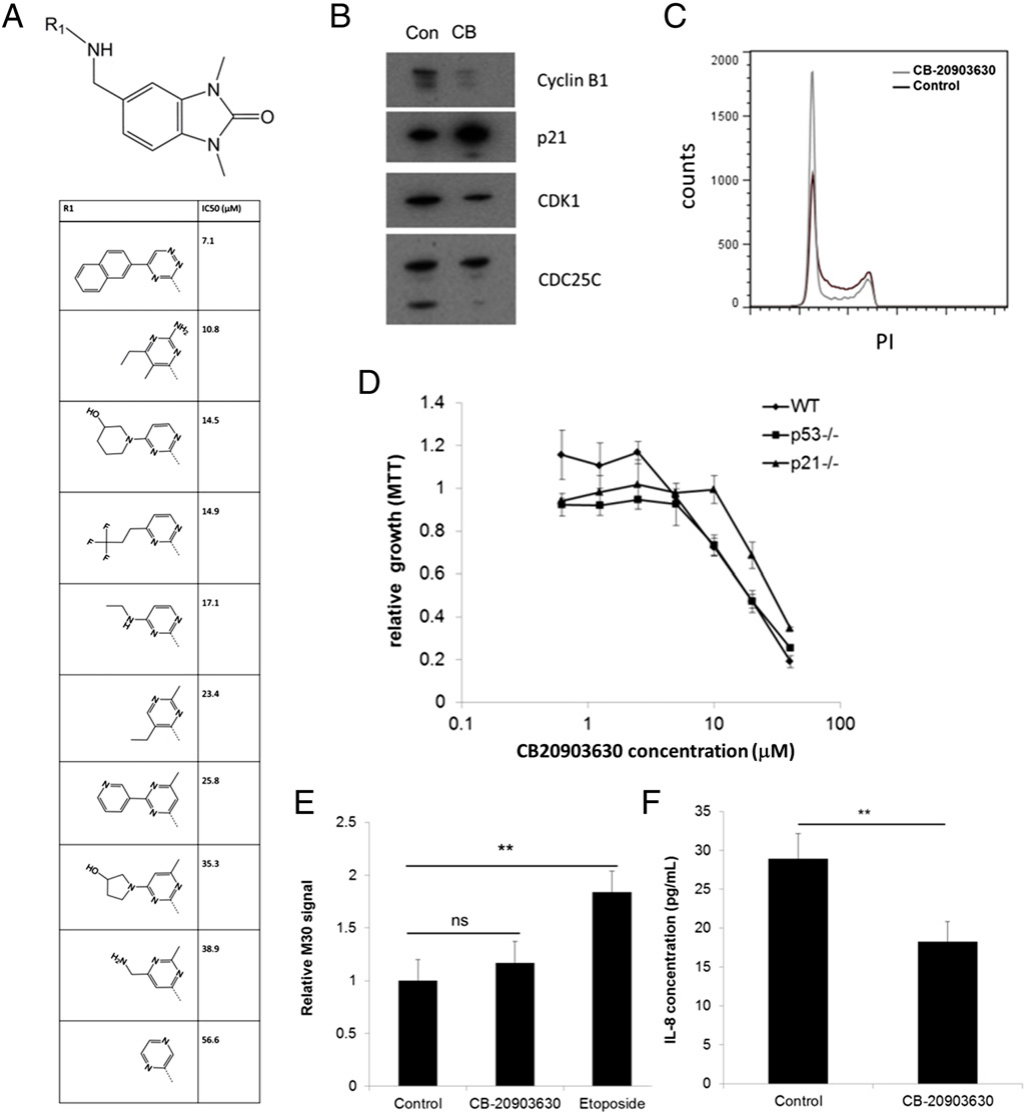
Structure-activity for the benzimidazolone scaffold and cell cycle effects of CB-20903630. (A) MTT SAR analysis of commercially available related analogues identified in the Chembridge catalogue. Mean IC50 of three experiments is shown. (B) Western blotting analysis of cell cycle effects in CB-20903630–treated HCT116 cells. Representative blots are shown. The experiment was performed twice. (C) Propidium iodide FACS analysis of cell cycle phase in control or treated cells. A representative histogram is shown. The experiment was performed three times. (D) MTT growth inhibition CB-20903630 dose-response in HCT116 or p53^−/−^ and p21^−/−^ isogenic variants. Mean ± SEM of three experiments. (E) Apoptosense CK18 assay of CB-20903630 or etoposide treatment in HCT116 cells. Mean ± SEM of three experiments (significance assessed by ANOVA: ns, not significant, **P < .01). (F) Suppression of IL-8 levels by CB-20903630 in HCT116 cells. Mean ± SEM of three experiments (significance assessed by ANOVA: **P < .01).

To test preliminary SAR around CB-20903630, we searched commercial vendor libraries, identifying close structural analogues with a range of lipophilicities and functionalities, and obtained 10 structurally related analogues (Figure 4*A*). There were few commercially available analogues which maintained the 4-methyl-6-cyclobutyl motif present in CB-20903630, so we instead focused on analogues retaining the benzimidazolone motif.

Within this set (compounds 101-110), we found a range of MTT growth inhibition activities, with compound 101, containing the 1,2,4-triazine naphthyl group, possessing a respectable IC50 of 7.1 μM, in line with CB-20903630. Reassuringly, additional changes on this portion of the molecules were also tolerated, with both saturated (compounds 103 and 108) and unsaturated (compound 107) being tolerated. In addition, the presence of basic (compound 109) and neutral (compounds 102-106, 108, and 110) functionality indicates that there is potential to further optimize this series.

Because CB-20903630 remained among the best of the set tested, its identity and purity (> 95%) were confirmed at resupply. We then investigated its effects on cell-cycle effectors involved in mediating arrest during senescence. HCT116 cells were treated for 48 hours in the presence of DMSO or 10 μM CB-20903630. Cells were harvested for Western blotting of cyclin B1, p21, CDK1, and CDC25C (Figure 4*B*). Cyclin B1, CDK1, and expression of the short isoform of CDC25C were all downregulated by CB-20903630 treatment, whereas levels of p21 were elevated (Figure 4*B*).

To confirm the cell cycle effects of the compound, we performed propidium iodide FACS analysis in treated HCT116 cells. Forty-eight–hour treatments at 20 μM were found to produce more robust effects than 10 μM, so we retained this dose for further growth-related assays. As shown in Figure 4*C*, CB-20903630 promoted a two-fold increase in G1 DNA content with a concomitant reduction in S-phase. Notably, there was no observed increase in the sub-G1 signal, suggesting that the compound does not significantly promote apoptosis and growth inhibition is primarily mediated through a G1 block.

We next compared growth inhibition of HCT116 with the isogenic deletion variants HCT116-p53^−/−^ and HCT116-p21^−/−^ (Figure 4*D*). The parental and the p53 deleted lines showed similar profiles, suggesting that p53 is not essential for cell cycle arrest by the compound. However, an approximately 1.5-fold reduction in sensitivity was observed in the p21 deleted cells. Hence, p21 but not p53 appears to play a role in the compound activity, in line with Figure 4*B*.

To confirm selectivity, we tested CB-20903630 in the M30 Apoptosense assay which measures an apoptotic neo-epitope of cleaved cytokeratin 18. CB-20903630 at 20 μM did not significantly increase cleaved CK18. However, the cytotoxic agent etoposide caused a 1.8-fold increase (Figure 4*E*, *P* < .01). Thus, CB-20903630 did not appear to induce substantial levels of apoptosis under these conditions in HCT116 cells.

Accelerated senescence is associated with an inflammatory phenotype characterized by secretion of a range of cytokines [6,7]. To investigate the inflammatory response of HCT116 cells, control or treated cell supernatants were tested in a multiplex assay analyzing the levels of 10 proinflammatory cytokines. Levels of most cytokines were low (Supplementary Figure S7) with the exception of IL8 (Figure 4*F*). CB-20903630 did not induce a proinflammatory signature. Indeed, the only significant change was reduction in IL8 levels. Hence, despite its cell cycle effects, CB-20903630 did not induce the “senescence-associated secretory phenotype.”

**Figure 5:**
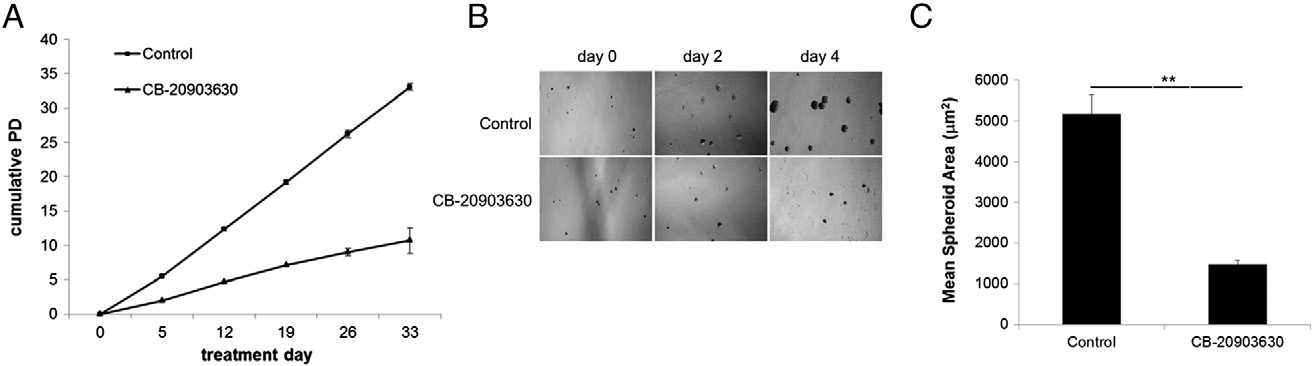
Long-term growth effects of repeat treatment with CB-20903630 and inhibition of multicellular spheroid growth. (A) HCT116 cells were maintained in culture and treated twice-weekly with CB-20903630 or vehicle. Cell numbers were counted weekly for calculation of cumulative population doublings. Mean ± SEM of three experiments. (B) HCT116 cells were adapted to serum-free conditions to generate a suspension line which grows as multicellular spheroids. Small spheroids were allowed to form in culture medium for 5 days then treated twice with CB-20903630 or vehicle. Representative micrographs obtained during the treatment period are shown. (C) Quantitation of mean area of 50 treated or control spheroids after 4 days of treatment with CB-20903630. Significance of population difference was assessed by Wilcoxon rank sum test (**P < .001).

We next treated HCT116 cells continuously with DMSO or 20 μM CB-20903630 twice weekly for 1 month to determine cumulative population doublings with weekly counting (Figure 5*A*). Control cells had undergone 33 population doublings by the end of treatment. In contrast, treated cell growth was severely retarded, and the cells underwent only 10.7 population doublings in total. Therefore, CB-20903630 treatment produced sustained inhibition of population growth.

We also examined CB-20903630 effects in an HCT116 spheroid model developed by adjustment to serum-free culture. Suspension cells were seeded for 5 days in the absence of treatment to allow initiation of multicellular spheroids, then swapped into 20 μM CB-20903630 or control medium (treatment day 0, Figure 5*B*), and cultured for a further 4 days. Treatment was repeated after 2 days. Compound was not removed between treatments.

Control spheroids significantly increased in volume in this period, whereas treated spheroids remained small (Figure 5*B*, *right panels*). Quantification of microscopic area of 50 individual spheroids in each condition showed a 3.5-fold difference (Figure 5*C*). Interestingly, following second treatment, many single cells were observed in treated flasks but not controls. In addition, treated spheroids were fewer in number and less tightly aggregated. It is possible that cell death pathways predominate under altered attachment.

### Expression Profiling and Pathway Analysis of CB-20903630 Activity

**Figure 6:**
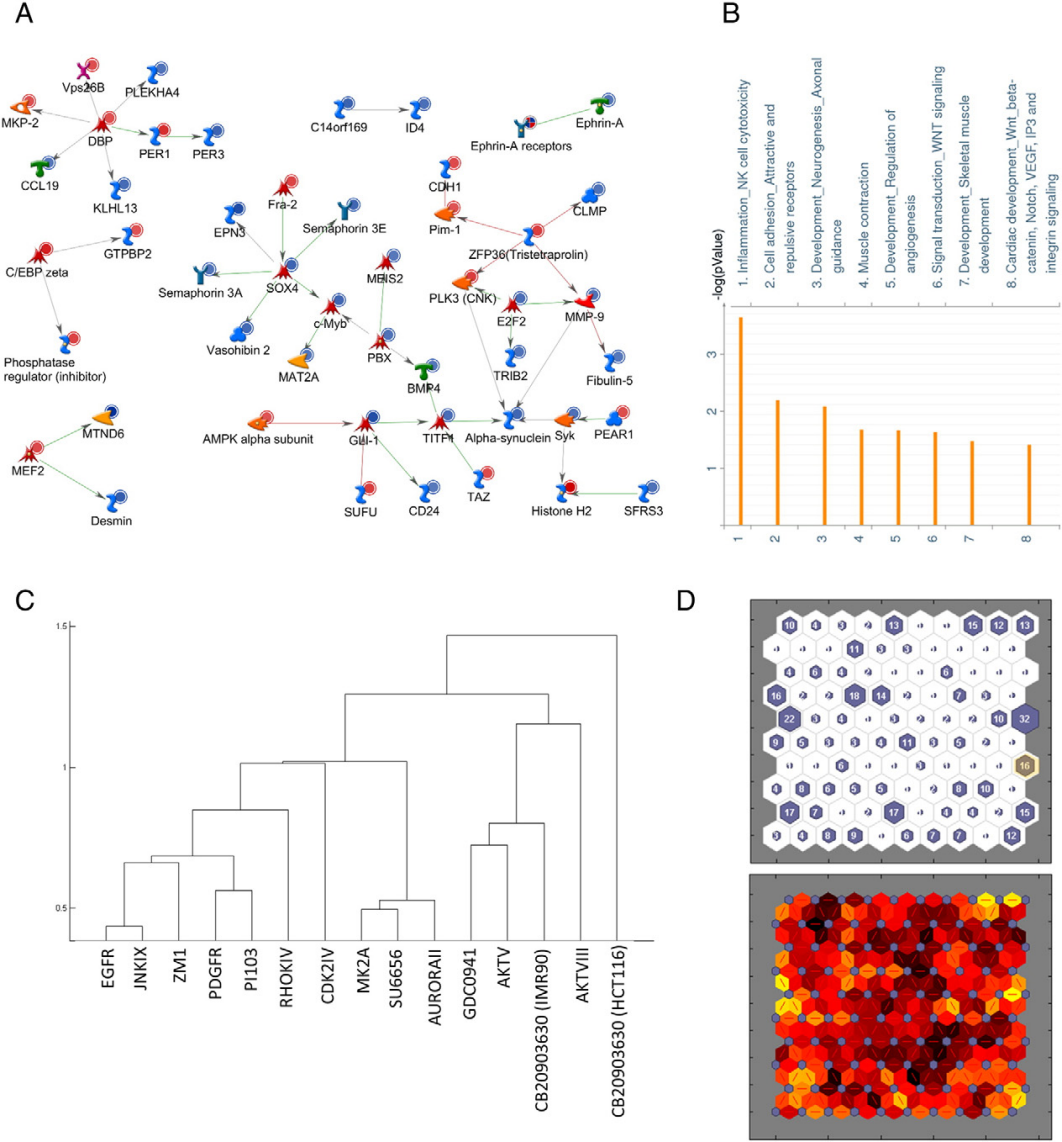
Microarray and structural analysis of CB-20903630. (A) Direct interactions network of differentially expressed genes in HCT116 cells treated with 10 μM CB-20903630. RNA samples from DMSO versus compound-treated cells were profiled on Agilent whole genome expression arrays. Differentially expressed gene lists were analyzed in MetaCore by the direct interactions algorithm to obtain the network model. Green and red arrows indicate known activating or inhibitory interactions between entities, respectively. Red and blue circles indicate upregulation and downregulation of expression relative to vehicle treatment, respectively. (B) Significant differentially affected GeneGo process networks under CB-20903630 treatment in HCT116 cells obtained by enrichment analysis of differentially affected genes. (C) Clustering of process network profiles with cumulative hypergeometric probability of pairwise overlap as the unweighted distance metric. (D) SOM structural clustering of CB-20903630 and known kinase inhibitors (see Materials and Methods section for source of comparator structures) after 200 training cycles. (Upper panel) Compound loadings: CB-20903630 and 15 other compounds loaded on the highlighted neuron in the upper panel. Numbers indicate number of compounds on each neuron. (Lower panel) Visualization of neighbor weights: the CB-20903630 neuron is not strongly clustered with its neighbors (darker bands indicate larger distances).

To investigate the mechanism of action of CB-20903630, we performed microarray analysis using cDNA from cells treated for 48 hours with DMSO or 10 μM CB-20903630. We identified differentially expressed transcript IDs with greater than three-fold intensity change (p < 0.05) between control and treated cells. Modeling in MetaCore [28] generated a network of known direct interactions among the differentially expressed genes. All direct interactions with cluster size ≥ 2 were included (Figure 6*A*).

Several transcription factors associated with development and proliferation were affected, including upregulation of Fra-2, DBP, and C/EBP, whereas E2F2, Gli-1, MEIS2, PBX, and Sox4 were downregulated. Interestingly, a number of secreted and membrane proteins were also downregulated including CCL19, vasohibin 2, semaphorins, fibulin-5, MMP9, BMP4, and ephrin A. These results suggest that the compound may regulate a secretory program distinct from the inflammatory markers investigated above. Clock genes PER1 and PER3 were also differentially regulated, in line with a previous study which found clock gene repression in vascular smooth muscle cells undergoing telomere-dependent senescence [36]. Process enrichment indicated that CB-20903630 promotes differential expression on inflammatory and developmental signaling networks as suggested by the model (Figure 6*B*).

CB-20903630 contains a kinase hinge-binding motif [37], indicating that the compound may target a cell cycle–related kinase. We generated expression profiles of IMR90 fibroblasts treated with 13 well-characterized kinase inhibitors (Supplementary Table S3) most of which induce a senescence-associated heterochromatin foci–like phenotype [49] and apoptosis and/or cell cycle responses. This data set represents a range of senescence effects induced by different pathway-specific agents in cells with an intact senescence response.

To compare the effects of CB-20903630, we also treated IMR90 with the compound and compared both HCT116 and IMR90 profiles with the other inhibitors. Responses were clustered on significance of overlap in affected MetaCore process networks. Numbers of significant process networks under each inhibitor treatment were used to generate hypergeometric probabilities for each pairwise comparison, which we used as an unweighted average distance metric (Figure 6*C*).

EGFR inhibitor clustered with JNKIX. Cell cycle pathways, cell adhesion, and developmental and cytoskeletal processes were affected by this group. Two AKT inhibitors (AKTV and AKTVIII) are present in the analysis, alongside two PI3K inhibitors (PI103 and GDC0941). AKTV/GDC0941 clustered and the CB-20903630 process network profile in IMR90 cells also clustered in this group. Adhesion, inflammation, development, and proteolysis processes are strongly represented in this group. The AKTVIII profile was also close to these in the analysis, whereas the other PI3K inhibitor PI103 was more closely related to the PDGFR inhibitor; both of these had very large process network profiles (52 and 59 networks affected, respectively). MAPK inhibitor MK2A clustered with Src-family inhibitor SU6656 and with AuroraII, primarily affecting DNA damage, cell cycle, and apoptosis processes.

The CB-20903630 process network profile in HCT116 clustered away from all others, possibly indicating that the compound mechanism affects different pathways in normal versus cancer cells. The profile in IMR90 appears to suggest similarity with agents targeting the PI3K/AKT pathway. CB-20903630 profiles in IMR90 and HCT116 were partially overlapping because five of the eight processes scoring as significant in HCT116 cells were also significant in IMR90 cells (Figure 6, *A* and *B*, and Supplementary Figures S8 and S9). Inflammatory processes were also highly represented in IMR90. However, IMR90 cells also scored highly in a range of development and proteolysis processes shared by the PI3K/AKT agents, making the observed profile more similar to these. Thus, different pathways may be affected by the compound in normal versus cancer cells.

To determine whether CB-20903630 is structurally related to existing kinase inhibitors, we performed clustering analysis on 3D pharmacophores comparing CB-20903630 alongside 527 known kinase inhibitors using a self-organizing map (Figure 6*D*). CB-20903630 loaded with 15 other compounds on a neuron which did not cluster strongly with neighbors. Examination of the structures showed prevalence of JNK2/3, VEGFR2, and GSK3 inhibitors (Supplementary File 2). However, CB-20903630 had little 2D similarity with these. Together, our results suggest that CB-20903630 is a selective cell cycle inhibitor which appears to be structurally novel.

## Discussion

Cellular immortality is a hallmark of cancer and a near-universal cancer target. However, recent clinical results suggest that telomerase may prove a more refractory target than had been hoped in solid tumors [38]. Multiple pathways regulate telomerase, and a variety of backup mechanisms may exist facilitating escape from inhibition [28,39]. On the other hand, strong interest in senescence induction as an alternative target to reverse limitless replicative potential of cancer cells has also emerged in recent years.

We began with a strategy to match training compound sets to the expression profile of telomere dysfunction. We identified a target panel, optimized a neural network ensemble, and screened a 2M-compound virtual library. Virtual hits were prioritized based on ADMET filters and pharmacophore clustering to identify a cell-based screening set, resulting in identification of CB-20903630. The compound promotes SA-β-gal in the majority of HCT116 cells and modulates cell cycle targets p21, cyclin B1, CDC25C, and CDK1, causing a G1 blockade without observable induction of cell death.

Preliminary SAR indicates scope for enhancement of CB-20903630 activity. However, relatively few close commercial analogues were found to be available, and we have therefore not exhaustively investigated this aspect and focused instead on the activity of CB-20903630. The growth inhibition effect appears to be in part dependent on p21 but not p53 based on sensitivities of isogenic HCT116 variants. In long-term treatments, the compound severely repressed population doubling times and strongly repressed growth in a multicellular spheroid model. The target of CB-20903630 is unknown. However, expression and structural clustering analysis suggest that the effects of the compound in normal cells have similarity with agents targeting the PI3K/AKT pathway, whereas in cancer cells, the effects diverged from other well characterized cell cycle inhibitors.

Virtual screening has previously been used for the identification of ligands for single targets [40]. In one recent example, the compound 6,6”-biapigenin was identified as a novel inhibitor of the NEDD8 activating enzyme (NAE) which is required for NEDDylation of a wide range of cellular targets. A previously identified NAE inhibitor showed broad activity against cancer cell lines, and the authors used molecular docking against a quaternary complex comprising the NAE subunits APPBP1 and UBA3 as well as its NEDD8 and ATP substrates to identify the new compound which showed low micromolar activity in Caco-2 cells [41]. Multitarget approaches such as that described here have not been widely investigated, although a recent study reported use of combinatorial support vector machine classifiers to identify dual-specificity ligands for a range of kinase pairs [42]. Furthermore, “inverse docking” in which individual compounds are docked against target panels has been suggested as a potentially powerful tool for compound repositioning strategies to complement existing pipelines in the pharmaceutical industry [43]. Our results suggest that virtual screening focused on target panels may also provide a useful approach for the identification of phenotype-focused libraries.

Ultimately, development of senescence therapeutics will require greater understanding of the regulation of senescence signaling networks. CB-20903630 is an interesting tool compound which appears to be a highly selective cell cycle inhibitor. The compound may therefore be a useful probe to identify new candidate markers and mechanisms associated with senescence and cell cycle responses. However, broad knowledge of the scaffold types that are able to regulate senescence pathways will also be required to identify a range of selective agents. In this paper, we identified a kinase-like scaffold by restricting our training set and library to “drug-like” chemical space. However, other regions of chemical space might also be worthy of consideration, such as natural product libraries or novel organometallic agents [44,45]. As in the current report, virtual screening might also provide an extremely useful tool to probe these novel library types in future studies.

## Materials and Methods

### Training Library Assembly and Neural Network Optimization

Training compounds were identified in confirmatory PubChem bioassay entries reported in Table 1 or from in-house data in the case of p21. All neural network optimization on neuron number, number of hidden layers, and training rule was performed using the Matlab neural network toolbox (Mathworks, Natick, MA). Ten-fold cross-validation was performed on each parameter set. The overall optimization workflow is shown in Supplementary Figure S2. Chemical descriptor sets were obtained using PaDEL Descriptor [33] and PowerMV [32].

Highly correlated descriptors and those with a variance of 0 were excluded, and the smaller representative set of descriptors was chosen using the Feature Selection option of the Canvas program [46,47] (Canvas, version 2.0; Schrödinger, LLC, New York, NY). 3D Pharmacophore fingerprints were also calculated in Canvas and ADME/Tox properties (Supplementary Table S2) in QikProp (QikProp, version 4.0; Schrödinger, LLC, New York, NY, 2014).

### Cell Lines and Compounds

The cells used were HCT116 colorectal cancer cells, their p53 and p21 deleted isogenic derivatives, and IMR90 fibroblasts. One hundred forty-seven virtual hits were selected based on the clustering analysis using 3D pharmacophore fingerprints. Compounds were initially sourced through E-Molecules (Stevenage, UK). CB-20903630 resupply and related analogues were obtained from ChemBridge (San Diego, CA). Structure was confirmed by nuclear magnetic resonance, and purity was confirmed by liquid chromatography/mass spectrometry. All other signal transduction inhibitors reported were obtained from Millipore (Supplementary Table S3). GRN163L was kindly provided by the Geron Corporation (Menlo Park, CA).

### MTT, Fluorescent SA-β-gal, and M30 Assays

For MTT assay, cells were treated twice over 48 hours with compounds and then incubated for a further 3 days before MTT assay (MTT supplied by Sigma, Dorset, UK). MTT reduction assays were performed using Softmax Pro software (Molecular Devices Ltd., Wokingham, UK). All experiments were repeated three times. Heat maps were generated in Tableau Desktop (Tableau Software, Seattle, WA). Fluorescent SA-β-gal assays were performed using the 96-well kit by Cell Biolabs (San Diego, CA). Cells were seeded overnight before 48-hour compound treatments. Five micrograms of protein was incubated in duplicate with assay buffer for 3 hours. Fluorescence was measured using a Safire plate reader (Tecan Group, Männedorf, Switzerland). All experiments were repeated twice. In colorimetric SA-β-gal staining assays, cells were fixed in gluteraldehyde and stained in the dark overnight with X-gal at pH 6. At least 500 cells in 5 random fields were counted for microscopic evaluation of proportions of stained cells in any treatment condition.

For M30 assay, cells were seeded in triplicate wells of 96-well plates overnight before addition of compound. Cells were treated for 2 days with compounds or vehicle then harvested. ELISA was performed on supernatants according the manufacturer’s instructions (VLVbio, Nacka, Sweden) with quantification using Softmax Pro software (Molecular Devices Ltd., Wokingham, UK). Experiments were repeated three times.

### Western Blotting

Twenty micrograms of protein was separated by SDS-PAGE, blotted onto polyvinylidene difluoride (Millipore, Watford, UK), and blocked overnight in PBS-T containing 5% nonfat dried milk. Antibodies were cyclin B1 (4135), p21 (2946), CDK1 (9112), and CDC25C (4688), all obtained from New England Biolabs UK (Hitchin, UK). Primary antibodies were detected with HRP-conjugated secondary. HRP was detected using ECL detection reagents (Amersham Pharmacia, Buckinghamshire, UK). Experiments were performed at least twice.

### FACS Analysis

Treated cells were fixed in 70% ethanol and stained with 0.05 mg/ml of propidium iodide solution containing 1 mg/ml of RNAse A (both obtained from Life Technologies, Paisley, UK) and 0.3% Tween-20 (Sigma, Dorset, UK). FACS was performed on a FACSverse instrument (BD Biosciences, Oxford, UK). Experiments were performed three times.

### Microarray Processing

RNA was labeled and amplified using the one-color microarray gene expression analysis protocol (Agilent Technologies, Santa Clara, CA), hybridized to Agilent whole human genome 4 × 44k Agilent whole human genome microarrays, and incubated for 17 hours at 60°C in a hybridization oven. Arrays were washed on a magnetic stirrer using Agilent wash buffers. Slides were scanned on an Agilent microarray scanner at 5-μm resolution, photomultiplier tube (PMT) gain at 100% and 10%. The extended dynamic range setting was corrected for saturation. Kinase inhibitor treatments in IMR90 were performed twice. CB-20903630 treatment of HCT116 was performed three times, but in IMR90 cells, only two independent experiments were performed because of compound availability. The data set has been submitted to the Gene Expression Omnibus with accession number GSE72621.

### Microarray Data Analysis

Microarray data were extracted using Agilent Feature Extraction software (Agilent Technologies, Santa Clara, CA). All array data were analyzed in GeneSpring for normalization and statistical analysis (Agilent Technologies, Santa Clara, CA). Intraarray normalization was carried out using the 75th percentile for each microarray. Significant differences in expression between control and treated cells were determined using unpaired *t* test. IDs with greater than three-fold intensity change, *P* < .05, were selected for further analysis.

### Process Profiles, Network Modeling, and Structural Clustering

Differentially expressed genes were analyzed in MetaCore (Thomson Reuters, New York, NY) using enrichment analysis by GeneGo process networks [27]. Probability of overlap was scored for each gene list tested against all process networks in the MetaCore database. Network analyses were performed using the GeneGo direct interactions algorithm [29]. For process network clustering, cumulative hypergeometric probability of pairwise overlap between process network profiles was calculated. Dendrograms were generated from the matrix of pairwise probabilities for all comparisons using unweighted average distances in Matlab (Mathworks, Natick, MA).

For structural clustering, 3D pharmacophores were calculated in Canvas. Included structures were CB-20903630 and 527 kinase inhibitors pooled from the Millipore Inhibitor Select 384-well panel (#539743) and from the GSK Published Kinase Inhibitor Set [48]. The finger prints were clustered on a 10 × 10 self-organizing map in Matlab using 200 training iterations. Compounds loading with CB-20903630 are given in Supplementary File 2.

## Statistical Analysis

All statistical analyses were performed in Microsoft Excel or Matlab.

## Figures and Tables

**Table 1:** Selected PubChem Bioassay Target Panel and Associated Compounds Identified as Relevant to Telomere-Dysfunction Process Network Profiles Generated in HCT116 Cells

Target	PubChem AID	Actives	Inactives	Description
p21	N/A	29	29	In-house screening data. Luciferase assay for activation of p21 promoter activity; inactives 50%-85% similarity with actives.
p53	624305	296	405	Confirmatory luciferase assay for activation of p53-dependent synthetic promoter reporter.
WEE1	1410	39	147	Increased WEE1-luciferase fusion gene activity; inactives 65% similarity to actives.
INCENP	473665	8	0	Small series of aurora inhibitors based on modification of an existing clinical candidate.
IL8	651758	38	88	Time-resolved FRET assay (IF) for IL8 secretion from cells; inactives 65% similarity to actives.
ATM	493192	41	36	Confirmatory ELISA for phosphorylation of ATM target protein.
MTORC1	2668	49	0	Confirmatory cell-based IF assay for phospho-rpS6.
HSP90	712	91	173	Confirmatory FP assay for HSP90 binding.
DNMT1	602386	179	21	Confirmatory fluorescein-labeled DNA oligomethylation assay.
BLM	2585	83	55	Confirmatory fluorescence quench DNA unwinding assay.
MDM2	1394	41	159	Confirmatory MDM2-luc autoubiquitination assay.
RECQL1	2708	173	321	Confirmatory fluorescence quench DNA unwinding assay.
SENP1	651697	117	60	Confirmatory kinetic FRET assay for SENP protease inhibition.
VDR	602201	159	115	Confirmatory FP assay for interaction of VDR and coregulator peptide.
EIF4E	855	77	486	Confirmatory TR-FRET for association of EIF4E/EIF4G.
RAD54	651657	394	63	Confirmatory fluorescent HR assay.
JMJD2A	488840	43	0	Confirmatory dissociation enhanced lanthanide fluorescence assay.

WEE1, homologue of S.Pombe Wee1; INCENP, Inner Centromere Protein; IL8, Interleukin 8; ATM, Ataxia Telangiectasia Mutated; MTORC1, Mammalian Target of Rapamycin Complex 1; HSP90, Heat Shock Protein (90kDa); DNMT1, DNA Methyl Transferase 1; BLM, Bloom Syndrome; MDM2, Mouse Double Minute 2 homologue; RECQL1, E.Coli RecQ Like helicase 1; SENP1, Sentrin Specific Protease family member 1; VDR, Vitamin D Receptor; EIF4E, Eukaryotic Translation Initiation Factor 4E; RAD54, homologue of S.Cerevisiae Rad54; JMJD2A, Jumanji Domain containing protein 2A; FRET, Fluorescence Resonance Energy Transfer; IF, immunofluorescence; ELISA, Enzyme Linked Immuno-Sorbent Assay; rpS6, Small Ribosomal Protein 6; FP, Fluorescence Polarization; TR-FRET, Time Resolved FRET; HR, Homologous Recombination.

## Appendix A Supplementary data

Supplementary File 1 Structures of the 147 screening compounds.

Supplementary File 2 Results of the Self Organizing Map analysis.
