# Supplementary material for: Identification of a Selective G1-Phase Benzimidazolone Inhibitor by a Senescence-Targeted Virtual Screen Using Artificial Neural Networks
Source: Neoplasia. 2015 Oct 19;17(9):704–15. doi: 10.1016/j.neo.2015.08.009 (PMC4611071; doi:10.1016/j.neo.2015.08.009)
Supplement: Supplementary file 3 — Supplementary Figures [file mmc3.docx]

Supplemetary Figures


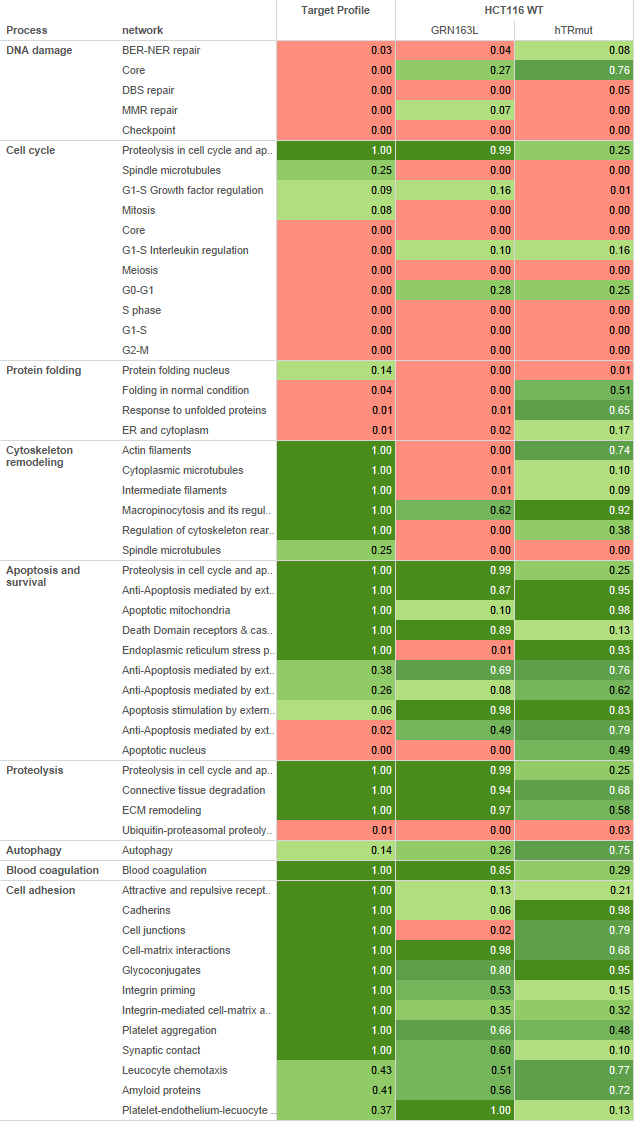


**Figure S1:** Comparison of process network profiles of target panel and telomere dysfunction stimuli. Red panels indicate significant enrichment in the process network of IDs in each list.


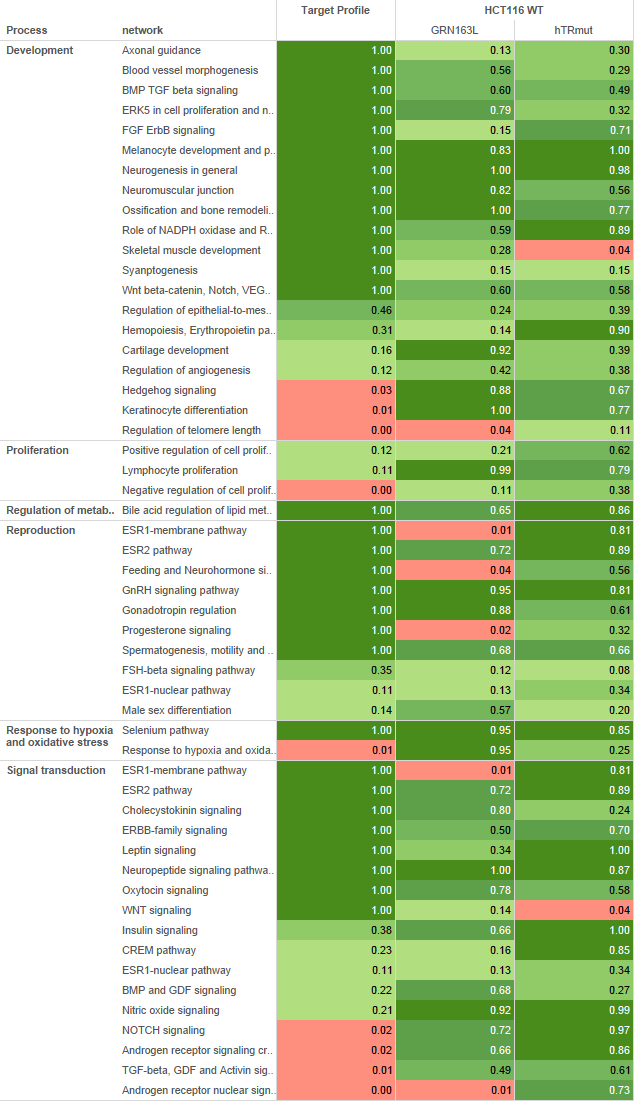


**Figure S1 (continued)**


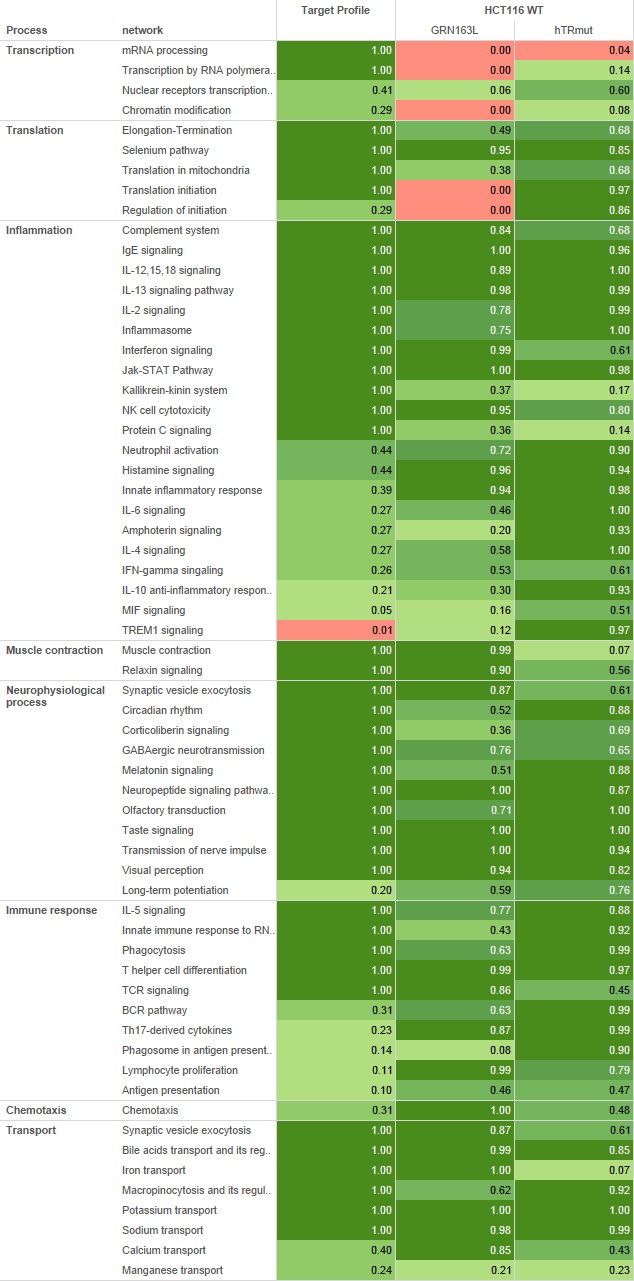


**Figure S1 (continued)**


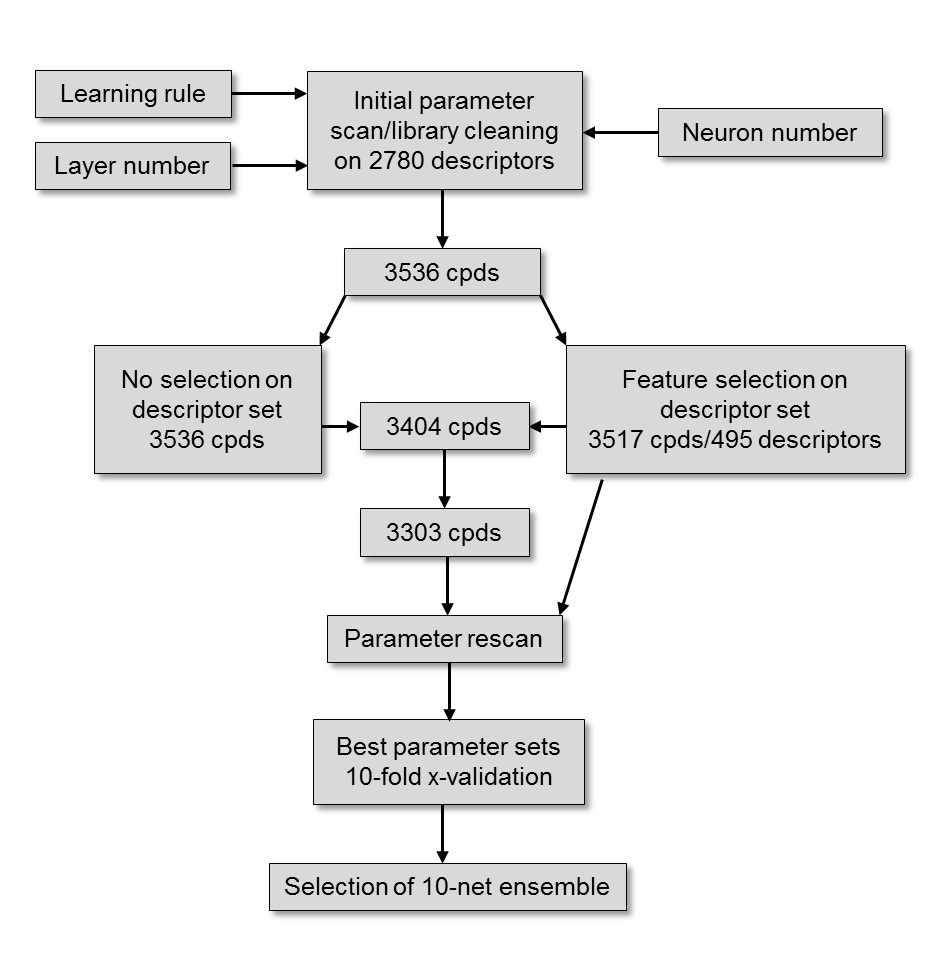


**Figure S2:** Overall network optimisation workflow.


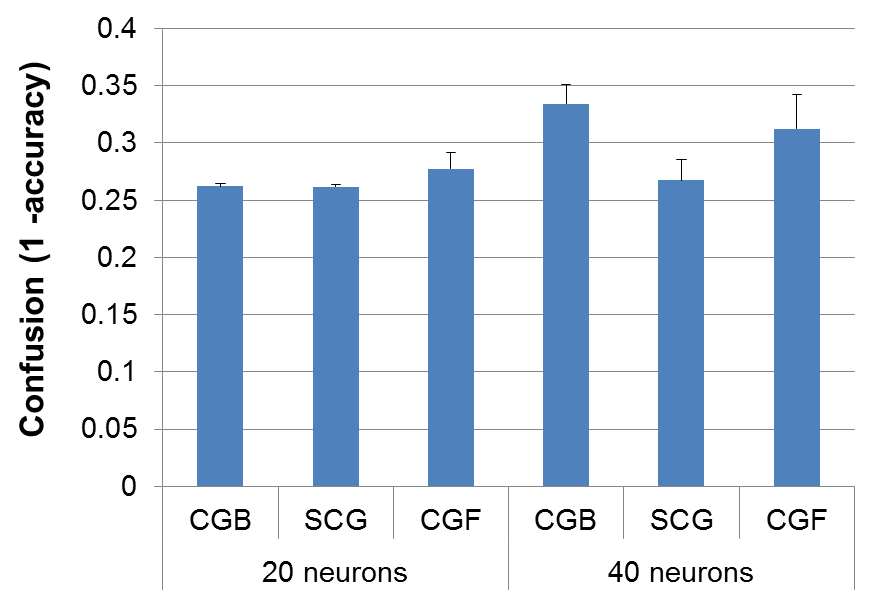


**Figure S3:** Initial ANN optimisation parameter scan using 3924 compounds and 2780 descriptors. Each parameter set was tested in 10-fold cross validation. Confusion values for the classifiers are shown. Best performance was achieved with 20 neurons in a single hidden layer using scaled conjugate descent. CGB, conjugate gradient with Powell-Beale restarts; SCG, scaled conjugate gradient; CGF, Fletcher-Reeves conjugate gradient.


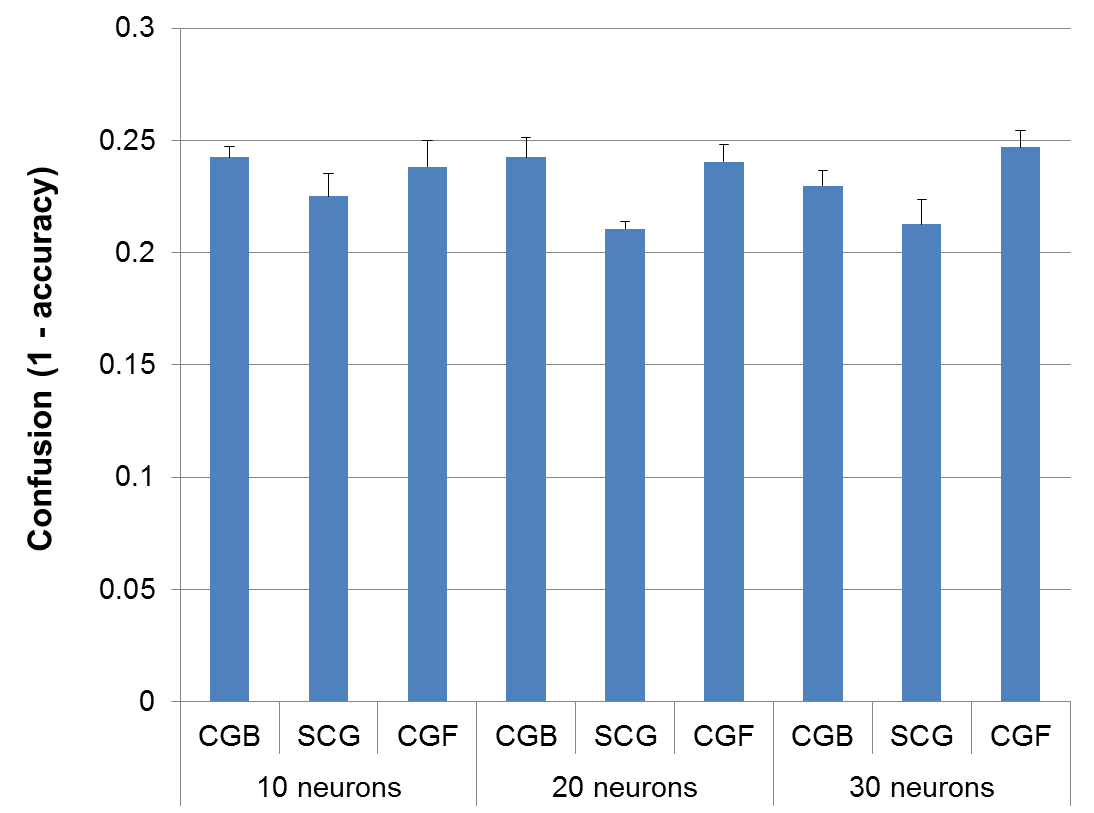


**Figure S4:** ANN optimisation parameter scan using 3517 compounds and 495 descriptors. Each parameter set was tested in 10-fold cross validation. Confusion values for the classifiers are shown. Best performance was achieved with 20 neurons in a single hidden layer using scaled conjugate descent. CGB, conjugate gradient with Powell-Beale restarts; SCG, scaled conjugate gradient; CGF, Fletcher-Reeves conjugate gradient.


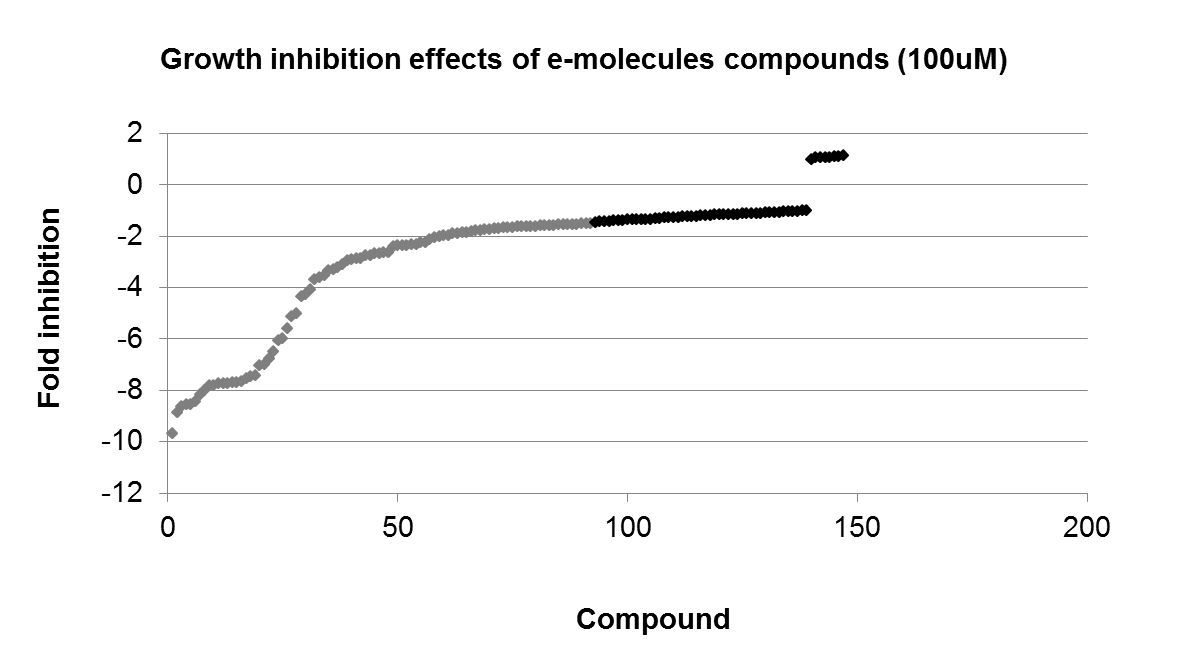


**Figure S5:** Single dose MTT growth inhibition results for 147 virtual hits. Compounds demonstrating at least 1.5-fold inhibition at 100μM were taken forward into dose response experiments (grey points).


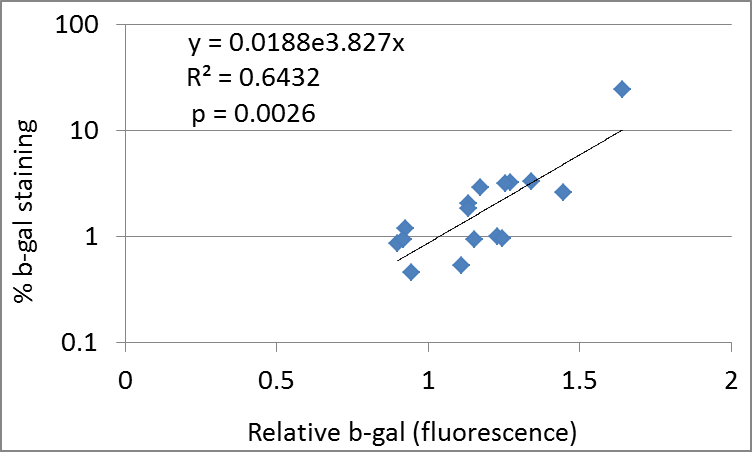


**Figure S6:** Log-linear relation between SA-β-gal staining and fluorimetric assays using 16 in-house cytotoxic compounds


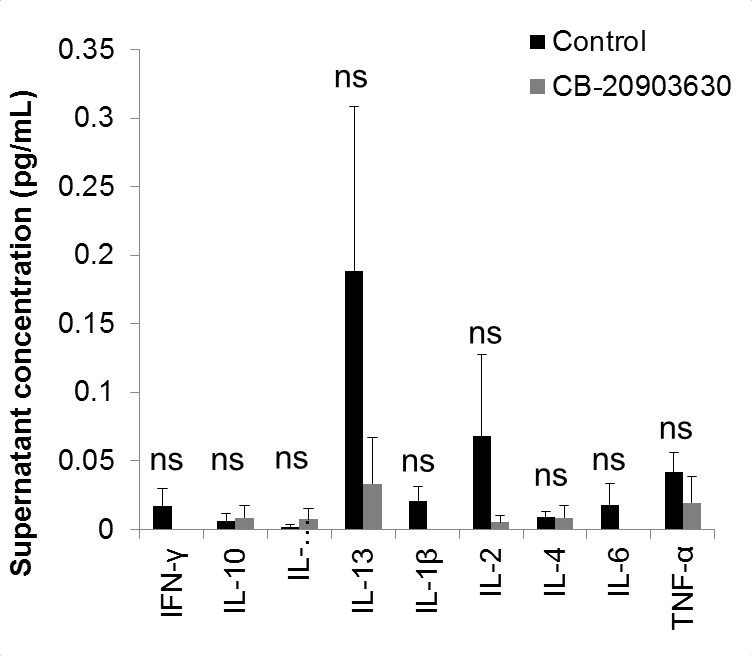


**Figure S7:** Multiplex pro-inflammatory cytokine sandwich assay in 20μM CB-20903630 treatment. Most cytokines were present in the cell culture supernatant at low concentration (some were below limits of detection in individual assays). No significant differences were observed except for IL-8 (main text).


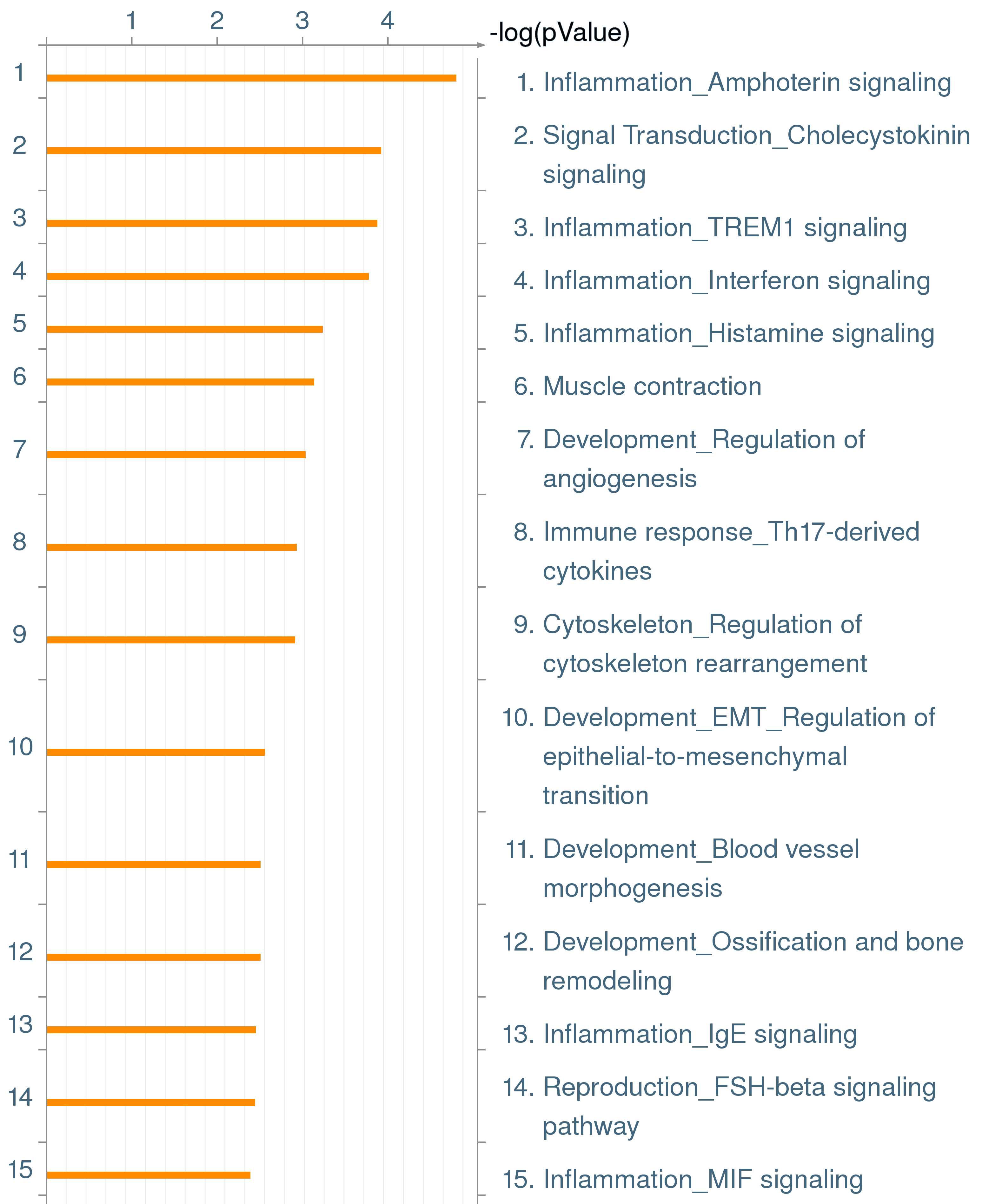


**Figure S8:** Significant differentially affected GeneGo process networks under CB-20903630 treatment in IMR90 cells obtained by enrichment analysis of differentially affected genes.


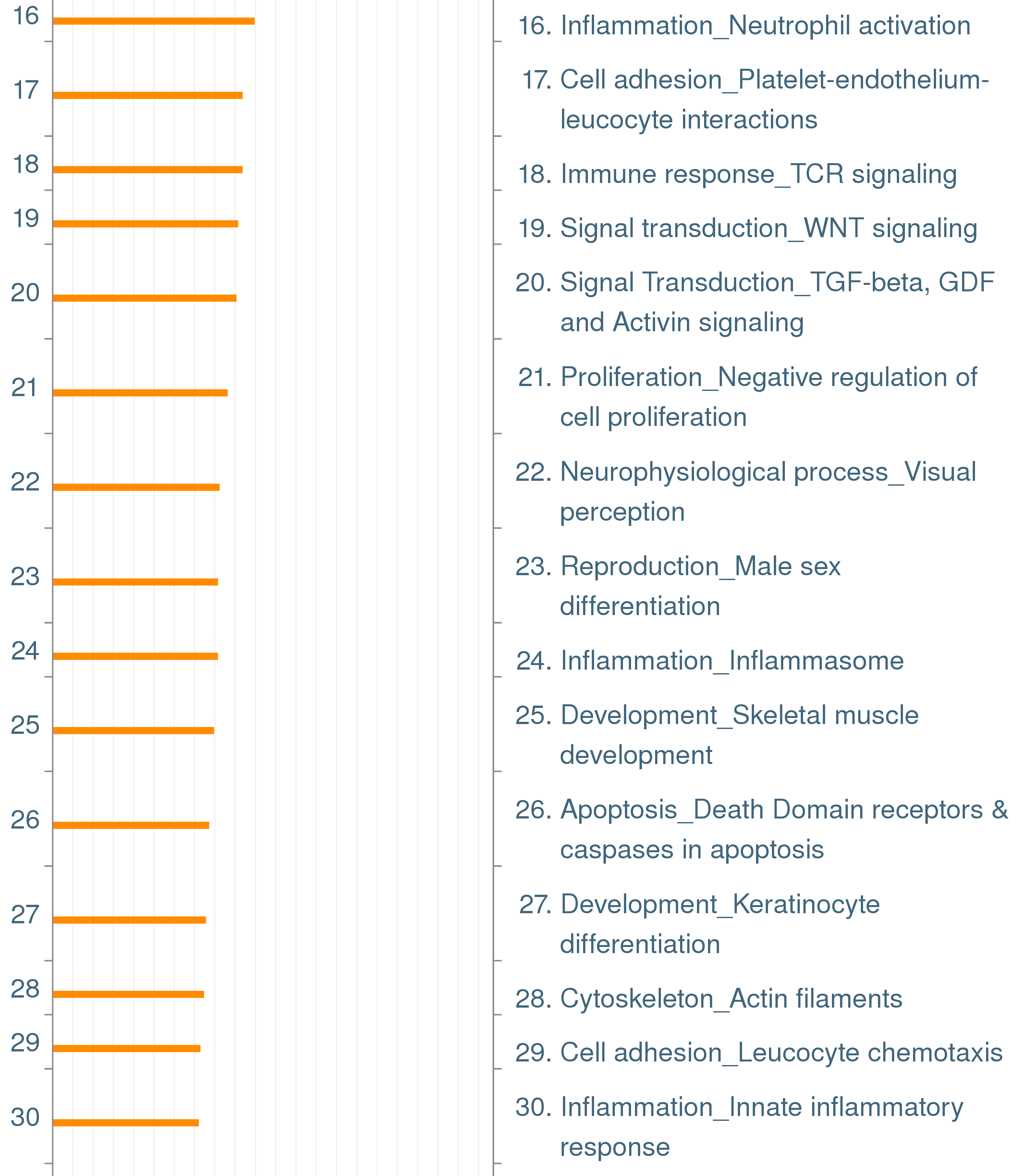


**Figure S8 (continued)**


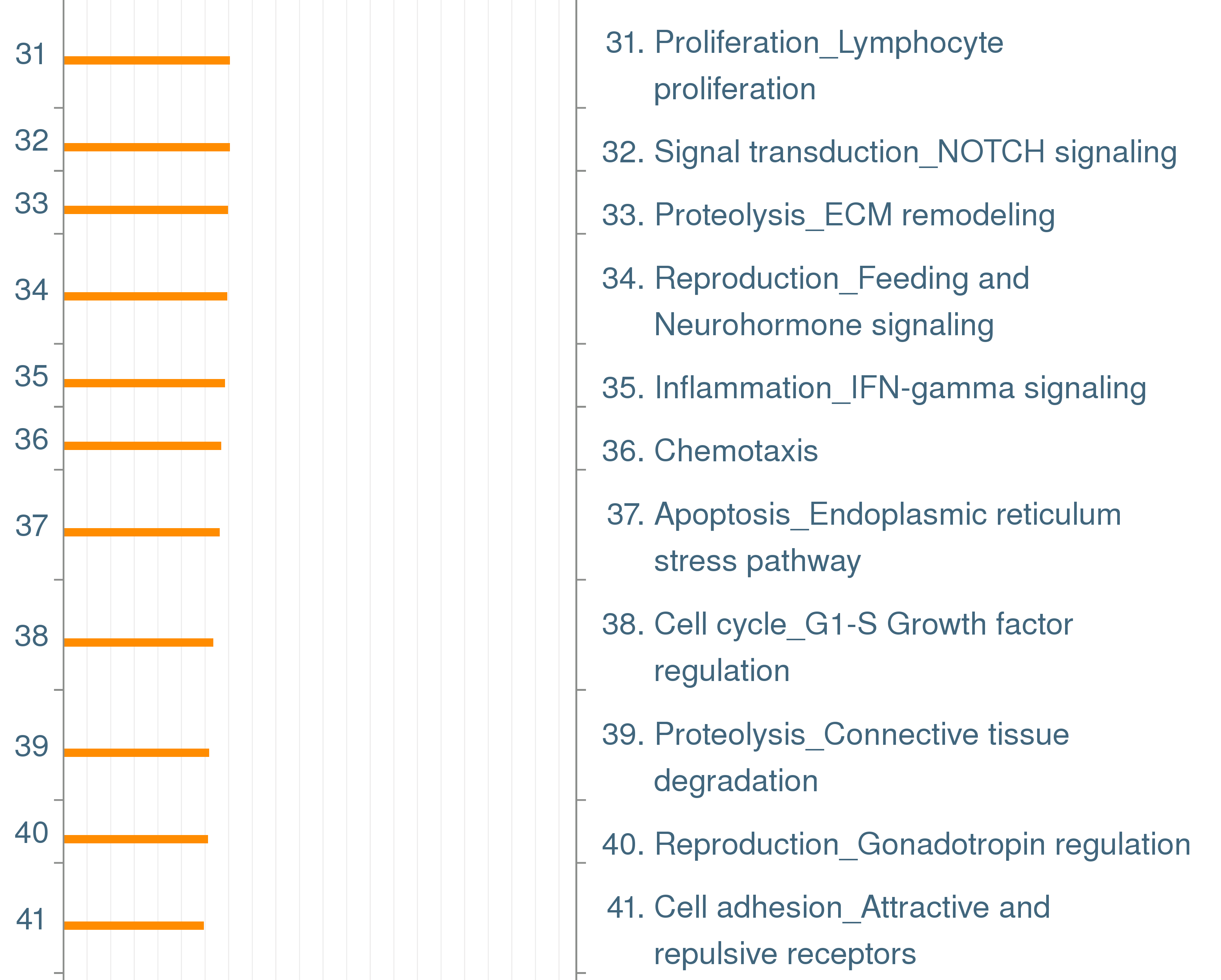


**Figure S8 (continued)**


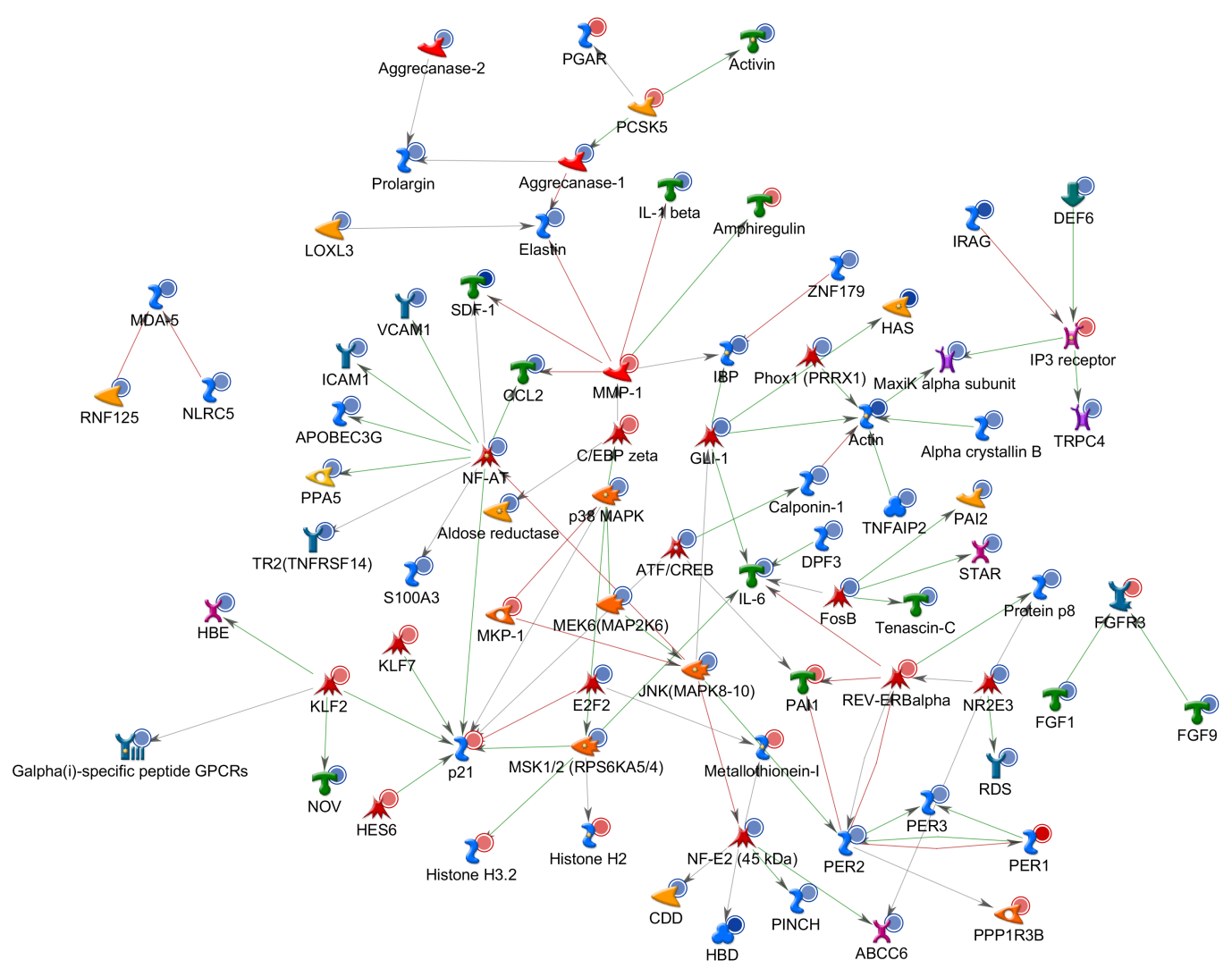


**Figure S9:** Direct interactions network of differentially expressed genes in IMR90 cells treated with 10μM CB-20903630. 2 independent treatments were analysed. Differentially expressed genes were analysed in MetaCore by the direct interactions algorithm. Green and red arrows indicate known activating or inhibitory interactions, respectively. Red circles indicate upregulation of expression relative to vehicle treatment.

Supplementary tables

| **Processes** | **Number of process networks** |
| --- | --- |
| Apoptosis and survival | 10 |
| Autophagy | 1 |
| Blood coagulation | 1 |
| Cell adhesion | 12 |
| Cell cycle | 11 |
| Chemotaxis | 1 |
| Cytoskeleton remodeling | 6 |
| Development | 20 |
| DNA damage | 5 |
| Immune response | 10 |
| Inflammation | 21 |
| Muscle contraction | 2 |
| Neurophysiological process | 11 |
| Proliferation | 3 |
| Protein folding | 4 |
| Proteolysis | 4 |
| Regulation of metabolism | 1 |
| Reproduction | 10 |
| Response to hypoxia and oxidative stress | 2 |
| Signal transduction | 17 |
| Transcription | 4 |
| Translation | 5 |
| Transport | 8 |

**Table S1:** GeneGo process network ontology used for enrichment analysis. Each process consists of a set of curated signalling networks.

| **Filter** | **Cutoffs** |
| --- | --- |
| MW | Range 300 - 450 |
| LogD | >= 0 |
| Predicted Caco2 apparent permeability | >= 400nm s^-1^ |
| Predicted IC50 hERG | >= 1µM |
| Predicted number metabolic reactions | <= 4 |

**Table S2:** Filters applied to the initial virtual hits.

| **Compound** | **Class** | **Targets** | **Millipore #** |
| --- | --- | --- | --- |
| EGFR | 4,6-dianilinopyrimidine | EGFR | 324674 |
| MK2A | *p*-amidophenol | MAPKAPK2a | 475863 |
| PDGFR | *bis* (1H-2-indolyl)-1-methanone | PDGFR/Flt-3 | 521230 |
| ZM1 | Quinazoline | Aurora A/B | 189410 |
| JNKIX | Thienylnaphthamide | JNK2/3 | 420136 |
| AKTVIII | Quinoxaline | AKT1/2 | 124018 |
| PI103 | Pyridinylfuranopyrimidine | DNA-PK/PI3K/mTOR | 528100 |
| CDK2IV | Purine | CDK2 < CDK1/4/5/7 | 238804 |
| GDC0941 | Thienopyrimidine | PI3K | 509226 |
| AKTV | Tricyclic nucleoside | AKT1/2/3 | 124038 |
| AuroraII | Anilinoquinazoline | Aurora A/B | 189404 |
| RhoKIV | Glycyl-isoquinolinesulfonamide | ROCK | 555554 |
| SU6656 | Indolinone | Src/Fyn/Yes/Lyn | 572635 |

**Table S3:** Signal transduction inhibitors used in process network profile clustering. All inhibitors were obtained from Millipore and the respective catalogue numbers are provided.
